# From Multiple Treatment Failures to Functional Improvement With Lower-Sodium Oxybate in Idiopathic Hypersomnia: A Case Report

**DOI:** 10.7759/cureus.115610

**Published:** 2026-09-01

**Authors:** Binh Pham

**Affiliations:** 1 Sleep Medicine, Southwest Healthcare Medical Education Consortium, Temecula, USA

**Keywords:** armodafinil, excessive daytime sleepiness, idiopathic hypersomnia, lower-sodium oxybate, methylphenidate, modafinil, multiple sleep latency test, polysomnography, solriamfetol, treatment-resistant hypersomnolence

## Abstract

Idiopathic hypersomnia (IH) is a chronic central disorder of hypersomnolence characterized by excessive daytime sleepiness despite adequate or prolonged sleep, with potentially substantial effects on occupational functioning, cognition, and driving safety. Pharmacologic management may be challenging when treatment is limited by inadequate efficacy or adverse effects. We report a young woman with severe hypersomnolence and a diagnosis of IH supported by clinical history and objective sleep testing. Multiple sleep latency testing demonstrated a mean sleep latency of one minute and 22 seconds, with no sleep-onset rapid eye movement periods (SOREMPs). Her Epworth Sleepiness Scale (ESS) score was 18/24, and symptoms included prolonged sleep duration, extended daytime naps, occupational impairment, and drowsy driving. Modafinil initially produced substantial benefit but subsequently lost efficacy despite dose escalation. Sequential trials of armodafinil, solriamfetol, mixed amphetamine salts, and methylphenidate were limited by tachycardia, inadequate efficacy or poor tolerability, paranoia, and persistent late-day sleepiness, respectively. Lower-sodium oxybate was subsequently initiated at 3 g once nightly but was reduced to 2.25 g because of significant nausea. With slower, individualized titration to 5.5 g once nightly, the patient reported substantial improvement in daytime sleepiness and functioning. Her ESS score decreased from 18/24 to 4/24, scheduled methylphenidate and caffeine were no longer required, and daytime napping was markedly reduced. This case illustrates the challenges of longitudinal pharmacologic management of IH and highlights the importance of individualized treatment selection, tolerability-guided oxybate titration, and assessment of functional outcomes beyond standardized sleepiness scores.

## Introduction

Idiopathic hypersomnia (IH) is a chronic central disorder of hypersomnolence characterized by persistent excessive daytime sleepiness (EDS) despite adequate sleep opportunity [1]. Beyond daytime sleepiness, the clinical phenotype may include prolonged nighttime sleep, severe difficulty awakening, sleep inertia, long and unrefreshing naps, cognitive dysfunction or “brain fog,” and persistent sleepiness despite substantial sleep duration [2]. These manifestations can be profoundly disabling, affecting academic and occupational performance, driving safety, and other aspects of daily functioning [3]. IH is therefore increasingly recognized as a heterogeneous disorder affecting the broader 24-hour sleep-wake period rather than as one defined solely by daytime sleepiness.

Diagnosis requires integration of the clinical phenotype, objective sleep testing, and exclusion of competing causes of hypersomnolence. The International Classification of Sleep Disorders (ICSD) framework requires chronic EDS that is not better explained by insufficient sleep, another sleep disorder, a medical or psychiatric condition, or medication or substance use, together with objective evidence of hypersomnolence and findings that do not fulfill the criteria for narcolepsy [1]. This distinction can be challenging because IH overlaps clinically with insufficient sleep, medication-related sleepiness, psychiatric disorders, and narcolepsy type 2 [2]. Overnight polysomnography (PSG), followed by the multiple sleep latency test (MSLT), is commonly used to exclude other sleep disorders and objectively assess daytime sleep propensity. However, MSLT findings in IH demonstrate limited test-retest reliability, reinforcing the importance of interpreting objective testing within the broader clinical context [4].

The functional burden of IH can be substantial, particularly when severe sleepiness persists despite prolonged sleep opportunity. Patients may experience impaired concentration, difficulty awakening for occupational or academic obligations, prolonged sleep requirements, a recurrent need for daytime naps, and safety concerns related to drowsy driving [3]. Compensatory strategies such as caffeine use and scheduled napping may be insufficient to maintain sustained wakefulness. Real-world studies have demonstrated broad effects of IH on employment, productivity, cognition, social functioning, and quality of life, emphasizing that treatment goals extend beyond reducing sleepiness to restoring sustained daytime function [3].

Management of IH is primarily pharmacologic and may require individualized treatment selection based on efficacy and tolerability. Modafinil is strongly recommended for adults with IH, whereas methylphenidate and sodium oxybate are conditionally recommended by the American Academy of Sleep Medicine [5]. Lower-sodium oxybate became the first pharmacologic treatment approved in the United States for adults with IH in 2021 and has demonstrated significant improvements in daytime sleepiness and overall IH symptom severity [6]. Treatment requires individualized dose optimization according to clinical response and tolerability, as adverse effects such as nausea commonly occur during titration [7].

Despite the availability of several pharmacologic options, some patients with IH experience incomplete or transient benefit, treatment-limiting adverse effects, or require multiple sequential medication trials before adequate symptom control is achieved [2,5]. We report a young woman with IH supported by objective sleep testing whose treatment course was limited by loss of efficacy with modafinil, tachycardia with armodafinil, inadequate efficacy and poor tolerability with solriamfetol, paranoia with mixed amphetamine salts, and persistent late-day sleepiness with methylphenidate. After transition to lower-sodium oxybate, treatment-limiting nausea required slow, tolerability-guided dose escalation. With individualized titration, her ESS score decreased from 18/24 to 4/24, scheduled methylphenidate and caffeine were discontinued, and daytime functioning substantially improved. This case illustrates the challenges of longitudinal pharmacologic management of difficult-to-treat IH and highlights the potential value of individualized, tolerability-guided titration when early adverse effects might otherwise limit treatment continuation.

## Case presentation

A 22-year-old woman with a history of major depressive disorder, generalized anxiety disorder, gastroesophageal reflux disease, migraine, and allergic rhinitis was referred by her neurologist for evaluation of EDS and concern for a central disorder of hypersomnolence. She reported excessive sleepiness beginning during her senior year of high school, with progressive worsening over the preceding two to three years. Her habitual sleep pattern before treatment was based on patient history rather than prospectively recorded sleep logs. On workdays, she typically slept from approximately 9:00 PM to 4:00 AM because of her schedule. On days off, she frequently slept until noon or 1:00 PM and subsequently took prolonged naps lasting approximately four to five hours. At its most severe, she reported sleeping for as much as approximately 18 hours within a 24-hour period. Despite these extended sleep periods, sleep was unrefreshing, and EDS persisted.

Her hypersomnolence resulted in substantial academic, occupational, and driving impairment. During nursing school, she frequently fell asleep during lectures and reported falling asleep when sitting quietly for approximately 30 minutes. At the time of presentation, she worked three 12-hour day shifts per week as a nurse and described significant difficulty maintaining wakefulness during her commute. She reported dozing while driving and used caffeinated beverages, music, and open vehicle windows to remain awake, although no motor vehicle collisions had occurred. The Epworth Sleepiness Scale (ESS), an eight-item questionnaire assessing the likelihood of dozing across common daytime situations, was used to quantify subjective daytime sleepiness [8]. Her baseline ESS score was 18/24. She denied cataplexy, sleep paralysis, and hypnagogic or hypnopompic hallucinations. There was no family history of hypersomnia or narcolepsy.

At the initial evaluation, she was 5 ft 2 in tall and weighed 155 lb, with a body mass index of 28.3 kg/m². Her blood pressure was 121/82 mmHg, pulse was 97 beats/minute, and oxygen saturation was 98% on room air. She appeared comfortable and well-nourished and was alert and fully oriented, with normal affect and judgment. Oropharyngeal examination demonstrated a Mallampati class II airway. Cardiopulmonary examination demonstrated a regular cardiac rhythm and clear lungs with unlabored respirations. Neurologic examination showed a normal gait, and no other pertinent abnormalities were identified.

A home sleep apnea test performed before the initial evaluation demonstrated an apnea-hypopnea index (AHI) of 3.8 events/hour and a minimum oxygen saturation of 73%, without evidence of obstructive sleep apnea based on the AHI. The low oxygen saturation nadir was not reproduced on subsequent attended PSG, which demonstrated a minimum oxygen saturation of 90% and no clinically significant sleep-disordered breathing. Given the severity of her EDS and concern for a central disorder of hypersomnolence, she subsequently underwent attended overnight PSG followed by a MSLT. PSG provides an objective assessment of nocturnal sleep and can identify sleep disorders that may contribute to EDS, whereas the MSLT assesses physiologic daytime sleep propensity across a series of nap opportunities and records the occurrence of sleep-onset rapid eye movement periods (SOREMPs). PSG demonstrated 412 minutes of total sleep time, a sleep efficiency of 87.4%, an AHI of 0.9 events/hour, a respiratory disturbance index (RDI) of 4.4 events/hour, and a minimum oxygen saturation of 90%, without evidence of clinically significant sleep-disordered breathing. The PSG findings are summarized in Table 1.

**Table 1:** Overnight polysomnography findings. The overnight polysomnogram demonstrated 412 minutes of total sleep time, with a sleep efficiency of 87.4%. The AHI was 0.9 events/hour, the RDI was 4.4 events/hour, the PLMI was 0.9 events/hour, and the minimum oxygen saturation was 90%, without evidence of clinically significant sleep-disordered breathing. AHI: Apnea-hypopnea index; PLMI: Periodic limb movement index; RDI: Respiratory disturbance index; REM: Rapid eye movement.

Parameter	Result
Total sleep time	412 minutes
Sleep efficiency	87.40%
Sleep latency	15.1 minutes
REM latency	125.5 minutes
AHI	0.9 events/hour
RDI	4.4 events/hour
PLMI	0.9 events/hour
Minimum oxygen saturation	90%

The following-day MSLT demonstrated sleep onset during all five nap opportunities, with individual sleep latencies ranging from 30 seconds to three minutes and 30 seconds. The mean sleep latency was one minute and 22 seconds, with no SOREMPs across the five nap opportunities. These findings demonstrated severe objective hypersomnolence without an MSLT pattern characteristic of narcolepsy and, when interpreted together with her clinical presentation and preceding PSG findings, supported a diagnosis of IH. The MSLT findings are summarized in Table 2.

**Table 2:** Multiple sleep latency test findings. The multiple sleep latency test demonstrated sleep onset during all five nap opportunities, with a markedly reduced mean sleep latency of one minute and 22 seconds. No sleep-onset rapid eye movement periods were observed across the five nap opportunities, consistent with severe objective hypersomnolence without an MSLT pattern characteristic of narcolepsy. MSLT: Multiple sleep latency test; SOREMP: Sleep-onset rapid eye movement period.

Nap	Sleep latency (min:sec)	SOREMP
1	0:54	No
2	0:30	No
3	3:30	No
4	0:30	No
5	1:30	No
Mean	1:22	N/A

The differential diagnosis included narcolepsy type 2, insufficient sleep syndrome, hypersomnia associated with a psychiatric or medical disorder, medication-related sleepiness, sleep-disordered breathing, and IH. Diagnostic interpretation was based on the International Classification of Sleep Disorders, Third Edition, Text Revision (ICSD-3-TR) framework. She met the chronicity criterion, with daily EDS present for several years, had no cataplexy, and demonstrated objective hypersomnolence on MSLT, with a mean sleep latency of one minute and 22 seconds and no SOREMPs. A two-week sleep diary completed immediately before PSG/MSLT documented variable sleep opportunity related to her work schedule, with shorter nocturnal sleep on several workdays but substantially longer sleep periods and additional daytime sleep episodes on days off. Therefore, some contribution from work-related insufficient sleep to the shortened MSLT latency could not be excluded, although insufficient sleep alone did not adequately explain the persistence of hypersomnolence during periods of extended sleep opportunity. Actigraphy was not performed. She had longstanding treatment with escitalopram and bupropion and used hydroxyzine nightly for anxiety. Before PSG/MSLT, she was advised to discontinue escitalopram and bupropion for two weeks; however, completion of the recommended washout could not be confirmed, and hydroxyzine use around the time of testing was not clearly documented. These uncertainties limit interpretation of the MSLT, particularly the absence of SOREMPs, and therefore reduce confidence in distinguishing IH from narcolepsy type 2, although the markedly shortened mean sleep latency still demonstrated severe physiologic sleepiness. Attended PSG demonstrated no clinically significant sleep-disordered breathing. The preceding home sleep apnea test had shown a minimum oxygen saturation of 73%, whereas attended PSG demonstrated a minimum oxygen saturation of 90%; the lower home-test value was not reproduced, and the available records did not establish whether it represented an artifact or a transient desaturation. Taken together, the chronic clinical phenotype, prolonged sleep when unrestricted, markedly shortened MSLT sleep latency, absence of cataplexy, and absence of clinically significant sleep-disordered breathing were consistent with IH, while variable pretest sleep duration and uncertain medication washout limited diagnostic certainty.

Following diagnosis, modafinil 200 mg daily initially produced substantial improvement, which she described as “life-changing,” but the benefit diminished after approximately two to three months. Despite escalation to 200 mg twice daily, her ESS remained 18/24, with persistent sleepiness at work and while driving and continued reliance on caffeine. Because of the initial response to daytime wake-promoting therapy, subsequent management continued to focus on alternative agents intended to sustain daytime wakefulness while balancing efficacy and tolerability. Armodafinil was discontinued because of tachycardia, solriamfetol provided inadequate benefit at 75 mg and was poorly tolerated at 150 mg, and mixed amphetamine salts were discontinued because of paranoia.

Methylphenidate 10 mg in the morning and 20 mg in the afternoon subsequently provided incomplete control of late-day sleepiness. During this treatment period, she was diagnosed with postural orthostatic tachycardia syndrome (POTS) and treated with propranolol 10 mg twice daily. She reported resting heart rates in the 120s without methylphenidate and in the 140s while taking it. Despite treatment, she continued to require caffeine and experienced severe sleepiness while driving home, including several episodes of swerving into adjacent lanes without a collision. Her prolonged sleep requirement also persisted; on days off, she slept until noon or 1:00 PM and subsequently took naps lasting approximately five hours.

Given persistent hypersomnolence and the limited efficacy or tolerability of multiple daytime wake-promoting therapies, treatment was transitioned to lower-sodium oxybate. Lower-sodium oxybate had been considered earlier in the treatment course; however, daytime therapies were initially prioritized because of their simpler administration and the patient's preference to pursue daytime treatment before initiating a nighttime oxybate regimen. As these therapies either lost efficacy or became limited by adverse effects, their clinical utility progressively diminished, shifting the balance of potential benefit and treatment burden in favor of lower-sodium oxybate.

Lower-sodium oxybate was initiated at 3 g once nightly. Once-nightly administration was used as an established dosing option for adults with IH; the available clinical records did not document a separate patient-specific rationale for selecting once-nightly rather than twice-nightly dosing. Significant nausea prompted dose reduction to 2.25 g once nightly, followed by gradual re-escalation according to tolerability. At 3.25 g once nightly, nausea had improved, and she subjectively estimated approximately 50% improvement in daytime sleepiness. Methylphenidate, which had been initiated before lower-sodium oxybate, remained in use during this early titration period because of residual afternoon sleepiness. Because more rapid dose escalation produced significant nausea, subsequent dose increases were spaced at approximately three-week intervals. This extended interval represented an individualized, tolerability-guided strategy rather than a standardized titration protocol. The available clinical records did not document each intermediate oxybate dose or the exact timing of methylphenidate discontinuation. By the most recent follow-up, after titration to 5.5 g once nightly, she had not required scheduled methylphenidate for approximately three months, although methylphenidate 10 mg remained available in the afternoon as needed.

At the most recent follow-up, at age 24, lower-sodium oxybate had been titrated to 5.5 g once nightly. She subjectively estimated approximately 80% improvement in daytime sleepiness, and her ESS had decreased from 18/24 to 4/24. The available clinical records did not reliably document the exact interval between initiation of lower-sodium oxybate and achievement of the 5.5 g dose or the duration at 5.5 g before the ESS score of 4/24 was recorded. Intermediate ESS measurements during oxybate titration were not available. She reported sustained daytime functioning, had not required scheduled methylphenidate for approximately three months, and no longer consumed caffeine. Napping was no longer necessary to maintain daytime function, with approximately one voluntary nap per week compared with prolonged obligatory naps earlier in her course. Her sleep schedule had consolidated to approximately 9:30-10:00 PM to 7:30 AM, and she denied enuresis, daytime grogginess, dizziness, or complex sleep behaviors. Although hydroxyzine had been used nightly for anxiety earlier in the clinical course, the available records did not clearly document whether it or another sedating medication was continued during lower-sodium oxybate treatment or whether specific concomitant-medication adjustments were made. Lower-sodium oxybate 5.5 g once nightly was continued.

Overall, the longitudinal treatment course demonstrated progressively constrained daytime pharmacotherapy because of inadequate efficacy and treatment-limiting adverse effects. Transition to lower-sodium oxybate, followed by individualized slow titration because of nausea, was associated with substantial subjective and functional improvement, a reduction in ESS from 18/24 to 4/24, cessation of caffeine use, a markedly reduced need for napping, and no requirement for scheduled methylphenidate for approximately three months. The complete pharmacologic treatment course is summarized in Table 3.

**Table 3:** Pharmacologic treatment course for idiopathic hypersomnia. Sequential wake-promoting and stimulant therapies were limited by loss of efficacy, inadequate response, treatment-emergent adverse effects, or a combination of these factors. Treatment changes were based on longitudinal clinical response, persistent patient-reported symptoms, ESS measurements where available, and adverse effects rather than on prespecified efficacy or discontinuation thresholds. Lower-sodium oxybate initially caused significant nausea, requiring dose reduction and individualized slower titration, but was subsequently associated with substantial subjective and functional improvement, including a reduction in ESS score from 18/24 to 4/24, cessation of caffeine use, a markedly reduced need for napping, and no requirement for scheduled methylphenidate for approximately three months. ESS: Epworth Sleepiness Scale; POTS: Postural orthostatic tachycardia syndrome.

Medication	Dose/regimen	Clinical response	Limitation or treatment outcome
Modafinil	200 mg daily, subsequently increased to 200 mg twice daily	Initially described as “life-changing,” with benefit lasting approximately two to three months; hypersomnolence subsequently recurred, with ESS remaining 18/24 despite dose escalation	Discontinued because of loss of sustained efficacy, with persistent hypersomnolence despite dose escalation
Armodafinil	Up to 250 mg daily	Trial discontinued	Discontinued because of treatment-emergent tachycardia
Solriamfetol	75 mg daily, subsequently increased to 150 mg daily	No meaningful improvement at 75 mg; 150 mg was poorly tolerated	Inadequate efficacy at 75 mg; 150 mg discontinued because of poor tolerability, with the patient reporting that she felt ill
Mixed amphetamine salts	10 mg in the morning and 20 mg later in the day	Persistent hypersomnolence	Discontinued because of treatment-emergent paranoia in the setting of persistent hypersomnolence
Methylphenidate	10 mg in the morning and 20 mg in the afternoon	Partial but incomplete control of late-day sleepiness, with continued caffeine use and drowsy driving	Incomplete efficacy, with persistent late-day sleepiness and driving impairment; treatment was additionally complicated by patient-reported resting heart rates in the 140s while taking methylphenidate compared with the 120s without methylphenidate in the setting of POTS
Lower-sodium oxybate	Initiated at 3 g once nightly; reduced to 2.25 g because of nausea, followed by gradual titration to 5.5 g once nightly	At 3.25 g, the patient subjectively estimated approximately 50% improvement in daytime sleepiness; at 5.5 g, she reported approximately 80% subjective improvement, her ESS score decreased from 18/24 to 4/24, and daytime functioning improved	Initial nausea required dose reduction and individualized slower titration; treatment was continued as tolerability improved and was subsequently associated with substantial symptomatic and functional improvement

## Discussion

This case illustrates the challenges of longitudinal pharmacologic management in IH when treatment is progressively constrained by inadequate efficacy and treatment-emergent adverse effects. Multiple daytime wake-promoting and stimulant therapies failed to provide sustained, adequately tolerated symptom control, while hypersomnolence continued to impair occupational functioning and driving safety. Transition to lower-sodium oxybate represented a shift toward a mechanistically distinct nighttime therapy [5-7]. During individualized, tolerability-guided titration, the patient reported substantial improvement in daytime sleepiness and functioning, with a reduction in ESS score from 18/24 to 4/24, cessation of caffeine use, a markedly reduced need for napping, and no requirement for scheduled methylphenidate for approximately three months. Although the temporal association cannot establish causality, the course illustrates the importance of reassessing treatment efficacy, tolerability, medication burden, and functional outcomes throughout the longitudinal management of difficult-to-treat IH.

Modafinil is strongly recommended by the American Academy of Sleep Medicine for adults with IH [5], and its early use in this patient initially produced substantial subjective benefit. Randomized controlled data similarly demonstrate improvement in objective wakefulness with modafinil in IH [9]. However, treatment response is heterogeneous, and longitudinal clinical experience demonstrates variable efficacy and the need for alternative or combination therapies in some patients [10]. In this case, the initial benefit diminished after approximately two to three months despite dose escalation, with persistent hypersomnolence and an ESS score of 18/24. This course demonstrates that a substantial early response may not translate into sustained symptom control and supports continued assessment of real-world functioning in addition to symptomatic response [5,9,10].

Subsequent daytime pharmacotherapy became increasingly limited by both efficacy and tolerability. Solriamfetol inhibits dopamine and norepinephrine reuptake [11], whereas methylphenidate can increase heart rate and blood pressure [12]. Cardiovascular considerations became particularly relevant after the patient was diagnosed with POTS. She reported higher resting heart rates while taking methylphenidate than when not taking it, although these observations occurred in the setting of established POTS and do not establish that methylphenidate alone caused the tachycardia. Modafinil has been studied in patients with POTS without significant worsening of standing heart rate compared with placebo [13]. In this patient, however, armodafinil was discontinued because of tachycardia, higher-dose solriamfetol was poorly tolerated, mixed amphetamine salts were discontinued after the development of paranoia, and methylphenidate provided incomplete control of late-day sleepiness while cardiovascular concerns persisted. Amphetamine-associated psychiatric adverse effects have also been described and provide clinical context for the development of paranoia during treatment [14]. Collectively, the diminishing efficacy and tolerability of successive daytime therapies favored consideration of a different therapeutic strategy.

Lower-sodium oxybate provided a pharmacologically distinct approach by targeting the nocturnal sleep period rather than relying exclusively on additional daytime wake promotion. Oxybate delivers gamma-hydroxybutyrate, which exerts central effects in part through gamma-aminobutyric acid type B receptor signaling, although the mechanism responsible for its therapeutic effects in IH remains incompletely understood. Controlled human neurophysiologic data demonstrate that gamma-hydroxybutyrate increases slow-wave sleep and enhances low-frequency sleep activity [15]. These findings provide a plausible framework through which modulation of nocturnal sleep physiology could influence subsequent daytime alertness, although a direct mechanistic relationship has not been established in IH. Independent clinical evidence supports the efficacy of lower-sodium oxybate in IH, with randomized controlled data demonstrating improvements in daytime sleepiness and overall symptom severity [6] and longer-term data supporting the persistence of benefit during continued treatment [16]. The clinical course observed here was consistent with these broader outcomes, although the uncontrolled nature of the case precludes attributing the patient's improvement solely to lower-sodium oxybate.

Dose optimization was an important practical feature of this patient's treatment course. Lower-sodium oxybate was initiated at 3 g once nightly, but significant nausea prompted reduction to 2.25 g, followed by gradual re-escalation according to tolerability. Clinical trial experience supports individualized lower-sodium oxybate titration based on efficacy and tolerability, with nausea among the commonly reported treatment-emergent adverse effects during dose optimization [7]. In this patient, recurrent nausea led to extension of subsequent dose increases to approximately three-week intervals. At 3.25 g once nightly, she subjectively estimated approximately 50% improvement in daytime sleepiness while still requiring methylphenidate for residual afternoon symptoms. Following further titration to 5.5 g once nightly, she estimated approximately 80% improvement, accompanied by a reduction in ESS score from 18/24 to 4/24 and elimination of scheduled methylphenidate use for approximately three months. The approximately three-week titration interval was a patient-specific strategy rather than an established titration protocol. These observations neither establish an individual dose-response relationship nor demonstrate that slower titration caused the subsequent clinical improvement; rather, tolerability-guided dose adjustment was temporally associated with continued treatment despite early nausea and later symptomatic improvement.

The improvement observed at follow-up extended beyond the ESS to several functional domains that had been substantially impaired earlier in the clinical course. Before oxybate treatment, the patient relied on frequent caffeine use, prolonged daytime naps, and scheduled stimulant therapy, yet continued to experience late-day sleepiness and clinically significant drowsy driving. At the most recent follow-up, she no longer used caffeine or scheduled methylphenidate, napping was no longer required to maintain daytime function, and she reported sustained daytime functioning. The available follow-up documentation did not specifically establish whether her earlier drowsy driving had completely resolved. This distinction is important because IH affects multiple domains beyond subjective sleepiness, including cognition, occupational functioning, and quality of life [3]. Treatment response should therefore be assessed not only with standardized sleepiness measures but also through changes in daily functioning, compensatory behaviors, medication burden, and safety-related outcomes.

Several limitations should be considered when interpreting this case. As a single-patient observation, the clinical response cannot establish causality or be generalized to other patients with IH. The reported approximately 50% and 80% improvements in daytime sleepiness during lower-sodium oxybate titration were subjective estimates rather than validated quantitative measures, although they were accompanied by an ESS reduction from 18/24 to 4/24 and meaningful functional changes. Diagnostic interpretation was also limited by uncertainty regarding pretest conditions. Although the patient was advised to discontinue escitalopram and bupropion for two weeks before PSG/MSLT, completion of the recommended washout could not be confirmed, and hydroxyzine use around the time of testing was not clearly documented. These uncertainties may have influenced sleep architecture and SOREMP expression and therefore reduce confidence in distinguishing IH from narcolepsy type 2 on the basis of the MSLT alone. In addition, the two-week pretest sleep diary demonstrated shorter sleep duration on several workdays, so some contribution from insufficient sleep to the markedly shortened MSLT latency cannot be excluded. However, the diary also documented substantially longer sleep periods and additional daytime sleep episodes when sleep opportunity was less restricted, consistent with the broader longitudinal history of persistent hypersomnolence despite prolonged sleep opportunity.

The diagnostic workup contained an additional unresolved discrepancy. The home sleep apnea test demonstrated a minimum oxygen saturation of 73%, whereas the subsequent attended PSG showed a minimum saturation of 90% without clinically significant sleep-disordered breathing. The available records did not establish whether the lower home-test value represented an artifact or a transient desaturation, and its clinical significance therefore remains uncertain. Longitudinal treatment assessment was also limited by the absence of intermediate ESS measurements, incomplete documentation of the exact timing of individual lower-sodium oxybate dose changes, and incomplete documentation of concomitant sedating-medication use during oxybate treatment. Moreover, stimulant exposure, caffeine use, sleep schedule, and management of POTS changed during the same general period as oxybate titration. Accordingly, the observed improvement was temporally associated with lower-sodium oxybate treatment but cannot be attributed exclusively to lower-sodium oxybate or to the individualized slower titration strategy. Finally, objective sleep testing was not repeated after clinical improvement, precluding assessment of whether the symptomatic response corresponded to measurable changes in physiologic sleepiness or sleep architecture. Future prospective studies should examine predictors of response to lower-sodium oxybate, relationships between dose optimization and functional outcomes, and whether individualized titration approaches influence treatment persistence among patients who experience early adverse effects.

## Conclusions

This case highlights the complexity of treating IH when multiple wake-promoting and stimulant therapies are limited by inadequate efficacy or adverse effects. Following loss of benefit with modafinil and subsequent limitations with armodafinil, solriamfetol, mixed amphetamine salts, and methylphenidate, transition to lower-sodium oxybate with individualized, tolerability-guided titration was temporally associated with substantial improvement in daytime sleepiness and functional status. The patient's ESS score decreased from 18/24 to 4/24, scheduled methylphenidate had not been required for approximately three months, caffeine use had ceased, and daytime napping was markedly reduced. However, concurrent changes in stimulant exposure, caffeine use, sleep schedule, and management of POTS preclude attributing the observed improvement exclusively to lower-sodium oxybate or to the slower titration strategy. This single case therefore does not establish comparative efficacy, causality, or an optimal treatment sequence but illustrates the importance of individualized medication selection, careful assessment of tolerability, and evaluation of functional outcomes throughout the longitudinal management of IH.

## References

[1] (2026). International Classification of Sleep Disorders (ICSD) (2023). https://aasm.org/clinical-resources/international-classification-sleep-disorders/.

[2] Trotti LM (2017). Idiopathic hypersomnia. Sleep Med Clin.

[3] Stevens J, Schneider LD, Husain AM, Ito D, Fuller DS, Zee PC, Macfadden W (2023). Impairment in functioning and quality of life in patients with idiopathic hypersomnia: the Real World Idiopathic Hypersomnia Outcomes Study (ARISE). Nat Sci Sleep.

[4] Trotti LM, Staab BA, Rye DB (2013). Test-retest reliability of the multiple sleep latency test in narcolepsy without cataplexy and idiopathic hypersomnia. J Clin Sleep Med.

[5] Maski K, Trotti LM, Kotagal S, Robert Auger R, Rowley JA, Hashmi SD, Watson NF (2021). Treatment of central disorders of hypersomnolence: an American Academy of Sleep Medicine clinical practice guideline. J Clin Sleep Med.

[6] Dauvilliers Y, Arnulf I, Foldvary-Schaefer N (2022). Safety and efficacy of lower-sodium oxybate in adults with idiopathic hypersomnia: a phase 3, placebo-controlled, double-blind, randomised withdrawal study. Lancet Neurol.

[7] Thorpy MJ, Arnulf I, Foldvary-Schaefer N (2022). Efficacy and safety of lower-sodium oxybate in an open-label titration period of a phase 3 clinical study in adults with idiopathic hypersomnia. Nat Sci Sleep.

[8] Johns MW (1991). A new method for measuring daytime sleepiness: the Epworth sleepiness scale. Sleep.

[9] Mayer G, Benes H, Young P, Bitterlich M, Rodenbeck A (2015). Modafinil in the treatment of idiopathic hypersomnia without long sleep time--a randomized, double-blind, placebo-controlled study. J Sleep Res.

[10] Anderson KN, Pilsworth S, Sharples LD, Smith IE, Shneerson JM (2007). Idiopathic hypersomnia: a study of 77 cases. Sleep.

[11] Baladi MG, Forster MJ, Gatch MB, Mailman RB, Hyman DL, Carter LP, Janowsky A (2018). Characterization of the neurochemical and behavioral effects of solriamfetol (JZP-110), a selective dopamine and norepinephrine reuptake inhibitor. J Pharmacol Exp Ther.

[12] Li L, Wang Y, Uppoor RS, Mehta MU, Farchione T, Mathis MV, Zhu H (2017). Exposure-response analyses of blood pressure and heart rate changes for methylphenidate in healthy adults. J Pharmacokinet Pharmacodyn.

[13] Kpaeyeh J Jr, Mar PL, Raj V (2014). Hemodynamic profiles and tolerability of modafinil in the treatment of postural tachycardia syndrome: a randomized, placebo-controlled trial. J Clin Psychopharmacol.

[14] Moran LV, Ongur D, Hsu J, Castro VM, Perlis RH, Schneeweiss S (2019). Psychosis with methylphenidate or amphetamine in patients with ADHD. N Engl J Med.

[15] Dornbierer DA, Baur DM, Stucky B (2019). Neurophysiological signature of gamma-hydroxybutyrate augmented sleep in male healthy volunteers may reflect biomimetic sleep enhancement: a randomized controlled trial. Neuropsychopharmacology.

[16] Morse AM, Dauvilliers Y, Arnulf I (2023). Long-term efficacy and safety of low-sodium oxybate in an open-label extension period of a placebo-controlled, double-blind, randomized withdrawal study in adults with idiopathic hypersomnia. J Clin Sleep Med.

